# The neurological wake-up test in severe pediatric traumatic brain injury: a long term, single-center experience

**DOI:** 10.3389/fped.2024.1367337

**Published:** 2024-02-23

**Authors:** Hilde D. Mulder, Jelte Helfferich, Martin C. J. Kneyber

**Affiliations:** ^1^ Department of Pediatrics, Division of Pediatric Critical Care Medicine, Beatrix Children’s Hospital, University Medical Center Groningen, University of Groningen, Groningen, Netherlands; ^2^ Department of Neurology, University Medical Center Groningen, University of Groningen, Groningen, Netherlands

**Keywords:** traumatic brain injury, pediatric, neurocritical care, wake-up test, NWT

## Abstract

**Objectives:**

To describe the use and outcomes of the neurological wake-up test (NWT) in pediatric severe traumatic brain injury (pTBI).

**Design:**

Retrospective single-center observational cohort study.

**Setting:**

Medical-surgical tertiary pediatric intensive care unit (PICU) in a university medical center and Level 1 Trauma Center.

**Patients:**

Children younger than 18 years with severe TBI [i.e., Glasgow Coma Scale (GCS) of ≤8] admitted between January 2010 and December 2020. Subjects with non-traumatic brain injury were excluded.

**Measurements and main results:**

Of 168 TBI patients admitted, 36 (21%) met the inclusion criteria. Median age was 8.5 years [2 months to 16 years], 5 patients were younger than 6 months. Median initial Glasgow Coma Scale (GCS) and Glasgow Motor Scale (GMS) was 6 [3–8] and 3 [1–5]. NWTs were initiated in 14 (39%) patients, with 7 (50%) labelled as successful. Fall from a height was the underlying injury mechanism in those seven. NWT-failure occurred in patients admitted after traffic accidents. Sedation use in both NWT-subgroups (successful vs. failure) was comparable. Cause of NWT-failure was non-arousal (71%) or severe agitation (29%). Subjects with NWT failure subsequently had radiological examination (29%), repeat NWT (43%), continuous interruption of sedation (14%) or intracranial pressure (ICP) monitoring (14%). The primary reason for not doing NWTs was intracranial hypertension in 59%. Compared to the NWT-group, the non-NWT group had a higher PRISM III score (18.9 vs. 10.6), lower GCS/GMS at discharge, more associated trauma, and circulatory support. Nine patients (25%) died during their PICU admission, none of them had an NWT.

**Conclusion:**

We observed limited use of NWTs in pediatric severe TBI. Patients who failed the NWT were indistinguishable from those without NWT. Both groups were more severely affected compared to the NWT successes. Therefore, our results may indicate that only a select group of severe pTBI patients qualify for the NWT.

## Introduction

Despite improvements in neurocritical care over the last decades, traumatic brain injury (TBI) remains a leading cause of mortality and significant morbidity. Intensive clinical monitoring, monitoring of intracranial pressure (ICP) and cerebral perfusion pressure (CPP), mechanical ventilation (MV), and continuous sedation are the main objectives of routine management of pediatric patients with severe TBI (1). This approach aims to prevent secondary injury by ensuring adequate brain perfusion and avoiding ischemia. The beneficial effects of sedation include decreasing intracranial pressure and cerebral oxygen consumption, thereby limiting the risk of secondary insults (1–3). However, accurate neurological assessment, which is considered to be the most fundamental clinical monitoring tool in TBI patients, is not possible in sedated patients. Reassessment of the patient's level of consciousness can detect neurological deterioration requiring prompt intervention (4).

Performing a neurological wake-up test (NWT) necessitates sedation interruption. This may be challenging especially in pediatric patients. Since short-acting sedatives such as propofol are contra-indicated in younger children, drugs with a relatively long half-life are used (5). In addition, sedation is also required to facilitate MV and reduce patient stress, which in turn may lead to episodic rises in cerebral blood volume (CBV) and ICP (6–8). This hampers the use of the NWT. Besides these practical considerations, the benefit of doing NWTs in especially severe TBI patients needs to be balanced against the risk of inducing a stress response. It has been suggested that not all patients can safely undergo NWTs due to potential secondary insults and the uncertainty about its clinical relevance in the context of advanced multimodality monitoring (9–14).

To our best of knowledge, there is no data on the usefulness feasibility of the NWT in neuromonitoring in severe pediatric TBI (pTBI). In fact, current international guidelines do not mention using the NWT (1, 15, 16). We therefore sought to characterize the use and outcomes of NWTs in severe pTBI patients admitted to our pediatric critical care unit (PICU).

## Patients and methods

### Study design and patient selection

We conducted an 11-year retrospective, observational, single-center study at a 20-bed PICU of the University Medical Centre Groningen (the Netherlands). Data from mechanically ventilated, sedated subjects ≤18 years with severe TBI [i.e., Glasgow Coma Scale (GCS) of ≤8] admitted between January 2010 and December 2020 was eligible for analysis. Subjects with non-traumatic brain injury were excluded. The Institutional Review Board approved the study and waived the need for informed consent (IRB UMCG #202200267).

### Patient management

Patients with moderate-to-severe TBI, admitted to our PICU are managed according to our protocol based on the most recent international guidelines (1, 17, 18), and with the following objectives: head elevation (30°), PaO_2_ > 10 kPa, PaCO_2_ between 4.5 and 6 kPa, targeted temperature <37.5°C, ICP <20 mmHg and CPP target between 40 and 50 mmHg (with infants at the lower end and adolescents at or above the upper end of this range). In the absence of invasive ICP monitoring a mean arterial pressure (MAP) ≥75th percentile per age is being pursued to maintain adequate CPP. If ICP increased, second tier of therapy using practical clinical algorithms included hyperosmolar therapies, mild hyperventilation, late application of hypothermia, barbiturate infusion or decompressive craniectomy surgery. Routine management of sedation includes using a combination of intravenous opioids and benzodiazepines or intravenous opioids and propofol, and neuromuscular blocking agents at the discretion of the bedside team.

### Collection of data variables

We collected baseline patient characteristics, information on trauma mechanism, presence of associated lesions, radiological examination within the first 24 h, information concerning neuromonitoring, neurosurgery, clinical and physiological data from the first 7 days of PICU admission. Radiological examinations were assessed by the attending radiologist. Ventilation time ≤24 h is described in actual hours, being easily deduced from the patients' records. When ventilation time exceeded 24 h, hours were simplified to whole day hours. The Pediatric RISk of Mortality [PRISMII]—24 h score and the Pediatric Index of Mortality [PIM] were calculated to assess patient status (19, 20). The GCS at onset was used to assess TBI and trauma severity (21).

### NWT

The NWT was defined as a sedation interruption allowing for a clinical examination by the attending pediatric neurologist within the first 24 h of PICU admission. The NWT was labelled as successful if the neurological evaluation could be completed without signs of increasing ICP allowing arousal or even detubation. Failure criteria for the NWT were defined as hemodynamic instability, respiratory distress, increasing ICP or other neurological distress. In addition, the NWT was also labelled as failed in the absence of any arousal [i.e., present or absent neurological deterioration (e.g., decreasing level of consciousness defined by a decrease in the GCS-M score of ≥2 points (16))].

### Outcomes of interest

The primary outcome of our study was the number of subjects in whom the NWT was performed, and to identify reasons why the NWT was not performed. Secondary outcomes included identifying which (type of) physician initiated the NWT, and the outcome of patients who underwent the NWT.

### Statistical methods

For analytical purposes, we stratified patients by NWT-outcome [i.e., NWT- success, NWT failure and non-NWT (i.e., NWT not performed)]. Categorical variables were described as absolute number and percentage (%) of total, and continuous variables as median and interquartile range [IQR]. Continuous variables were analyzed with the Mann-Whitney U test and Kruskal–Wallis test, categorical data was analyzed using the *χ*^2^ test (Fisher exact test if the value of any cell was <5). All analyses were performed with SPSS v23.0 [IBM Statistical Package for the Social Sciences (SPSS) for Windows, Armonk, NY: IBM Corp]. *P* values < 0.05 were accepted as statistically significant.

## Results

### Study population

**Figure 1:**
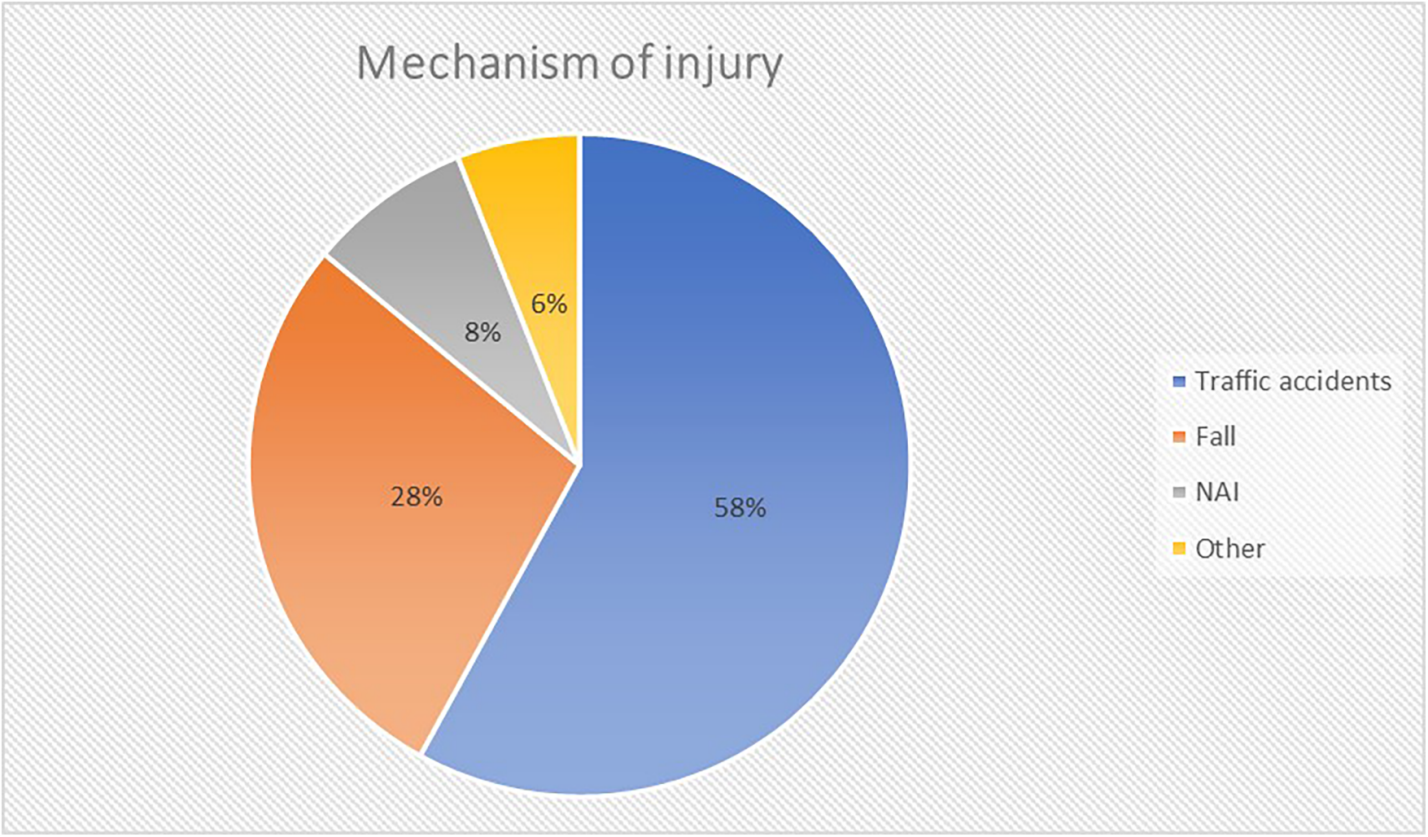
Mechanism of injury of enrolled patients. NAI, non-accidental injury.

**Figure 2:**
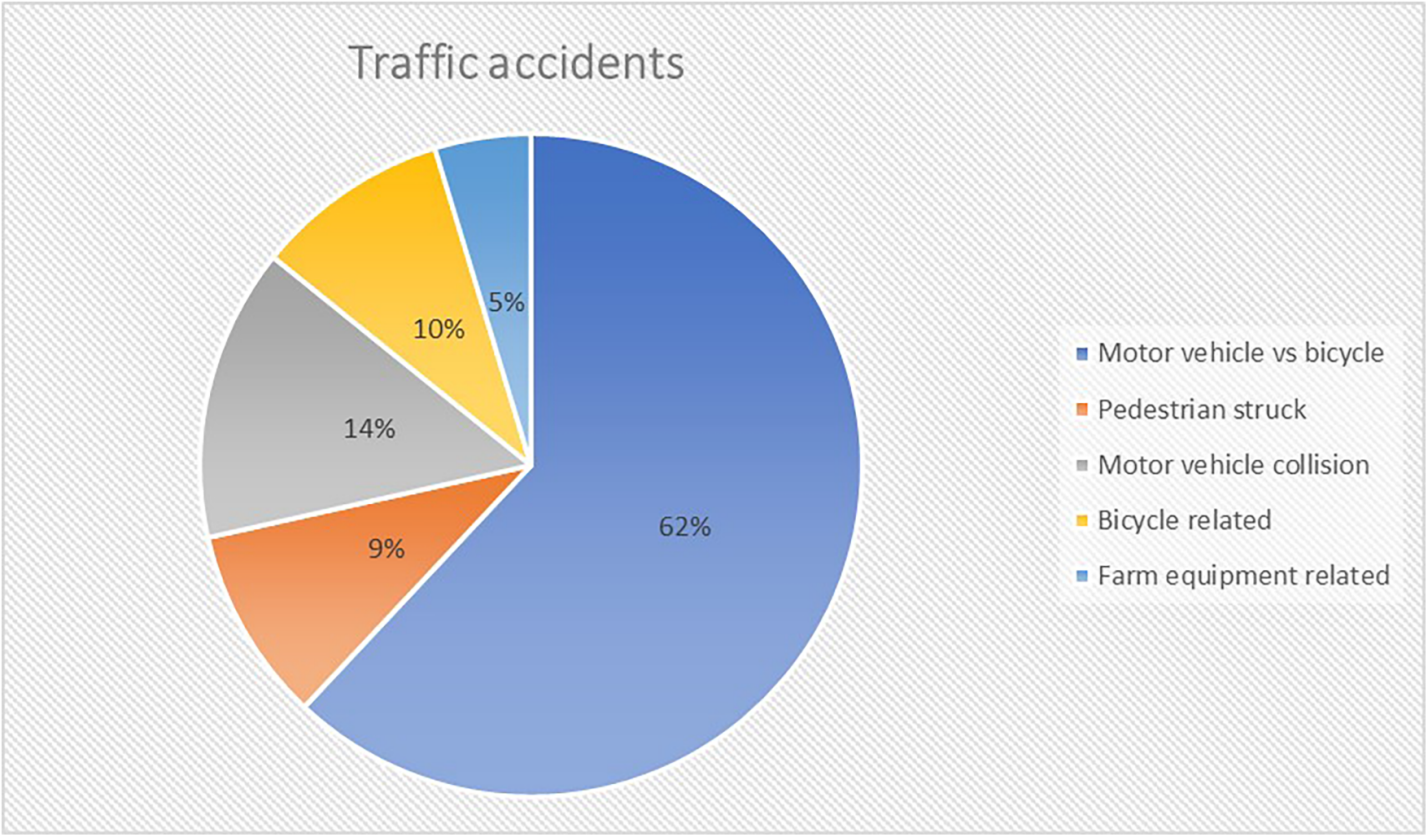
Subdivision of traffic accidents.

Out of 168 TBI patients admitted, 39 (23%) had a GCS ≤8. Subsequently, a further three patients were excluded: one who with anoxia following cardiac arrest and two patients who were not mechanically ventilated. Thus, data from 36 patients (21 male gender; 58%) with a median age of 8.5 years [2 months to 16 years] was available for analysis (Supplementary Table A). Median PRISM and PIM score was 14 [2–48] and −2.798 [−3.468 to 2.671]. The most common mechanism of injury were traffic accidents (TA; 58%) (Figures 1, 2). Median initial GCS and GMS was 6 [3–8] and 3 [1–5].

### NWT

NWTs were performed in 14 patients (39%), with seven (50%) labelled as successful (Table 1). NWTs were requested by either the pediatric intensivist, neurologist or neurosurgeon. Assessing awareness (57%) or neurological deterioration (43%) were mentioned as NWT-indications. The distribution of NWTs during the study period was equal (Supplementary Table B). No intracranial pressure monitoring was present at the time of the first NWT in any of the 14 patients.

**Table 1:** Descriptive characteristics of patients with or without NWT.

No	NWT	TM	iGCS	Indicator	Reason (non) test	Result	Survival	Ventilation [hours]	ICPm	CCT 1st 24 h
1	No	TA	5	NA	ND	–	Yes	264	No	Yes
2	Yes	Fall	5	PICU	RD	Awake	Yes	24	No	No
3	Yes	Fall	4	PICU	RD	Awake	Yes	12	No	No
4	Yes	TA	4	Neuro1	Aware	Not awake	Yes	72	No	Yes
5	No	TA	6	NA	ICH	–	Yes	168	Yes	Yes
6	Yes	TA	4	?	Aware	Disturb	Yes	120	No	No
7	No	TA	4	NA	ICH	–	No	24	Yes	No
8	No	TA	3	NA	ICH	–	Yes	816	Yes	Yes
9	No	O	3	NA	ICH	–	No	168	Yes	Yes
10	Yes	Fall	8	Neuro2	Aware	Awake	Yes	24	No	No
11	No	NAI	3	NA	ICH	–	No	168	No	No
12	Yes	TA	7	Neuro1	Aware	Not awake	Yes	1,056	Yes^a^	No
13	No	TA	7	NA	ND	–	Yes	240	No	Yes
14	No	NAI	3	NA	ICH	–	No	24	No	No
15	Yes	TA	4	Neuro1	Deter	Not awake	Yes	216	No	No
16	No	TA	3	NA	HDi	–	No	12	No	No
17	Yes	Fall	7	?	Aware	Awake	Yes	3	No	No
18	Yes	Fall	8	PICU	Aware	Awake	Yes	8	No	No
19	No	O	6	NA	ICH	–	Yes	240	Yes	Yes
20	No	NAI	6	NA	SE	–	Yes	216	Yes	Yes
21	Yes	Fall	6	?	Aware	Awake	Yes	6	No	No
22	Yes	TA	8	Neuro1	Aware	Not awake	Yes	96	No	No
23	No	Fall	8	NA	ND	–	Yes	48	No	No
24	Yes	TA	7	Neuro1	Aware	Not awake	Yes	192	No	No
25	No	TA	7	NA	ICH	–	Yes	480	Yes	Yes
26	No	TA	6	NA	ICH	–	Yes	192	Yes	Yes
27	No	TA	3	NA	ICH	–	Yes	240	No	Yes
28	Yes	TA	6	Neuro1	Deter	Disturb	Yes	336	No	Yes
29	No	TA	4	NA	p/o PICU	–	Yes	96	Yes	Yes
30	No	TA	7	Neuro1	p/o PICU	–	Yes	48	No	Yes
31	No	Fall	8	NA	ICH	–	No	12	No	Yes
32	No	Fall	7	NA	ICH	–	Yes	168	Yes	No
33	Yes	Fall	7	?	Aware	Awake	Yes	12	No	No
34	No	TA	3	NA	HDi	–	No	4	No	No
35	No	TA	3	NA	ND	–	No	6	Yes	No
36	No	TA	3	NA	ICH	–	No	24	No	No

Aware, awareness; CCT, computed tomography of head; Deter, deterioration; Disturb, neurological disturbance; HDi, hemodynamic instability; ICH, intracranial hypertension; ICPm, intracranial pressure monitoring; iGCS, initial Glasgow Coma Scale; NA, not asked; NAI, non-accidental injury; ND, not discussed; Neuro1, neurologist; Neuro2, neurosurgeon; No, number; NWT, neurological wake-up test; O, other; RD, reasonable detubation; SE, status epilepticus; TA, traffic accident; MoI, mechanism of injury.

^a^ After NWT.

Patients in whom the NWT was successful were significantly younger (5 [2–6] vs.12 [9–14] years (*p* = 0.008) and all had a fall from height as injury mechanism compared with all patients having a TA in the failure group, (*p* = 0.001) (Table 2). Overall GCS and GMS scores at onset were similar, but patients in whom the NWT was stopped had significant lower GCS/GMS scores at discharge PICU (12/5 vs.15/6, *p* = 0.010/*p* = 0.009). Patient characteristics were similar for associated trauma, initial cerebral computed tomography (CCT)-scan abnormalities, convulsions, circulatory support or survival. Patients in whom the NWT was successful were extubated shortly hereafter. They had a significantly shorter MV duration (12 [6–24] vs. 192 h [96–336] (*p* = 0.002) compared to the failed NWTs.

**Table 2:** Overall comparison between NWT-success and NWT-failure group.

*N*	NWT-success	NWT-failure	*p*-value
	7	7	
Age			
-year	−5[2; 6]	−12[9; 14]	0.008
Gender, male	2 (29%)	5 (71%)	0.286
PRISM	8.5 [5.0; 13.5]	13 [5; 17]	0.283
PIM	−3.0 [−3.1; −2.8]	−3.1 [−3.4; −2.9]	0.142
iGCS	7 [5; 8]	6 [4; 7]	0.393
iGMS	4 [3; 4]	4 [2; 5]	0.893
GCS aDP	15 [15]	12 [9; 12]	0.010
GMS aDP	6 [6]	5 [4; 6]	0.009
Survival	7 (100%)	7 (100%)	^a^
Initial CCT-scan abnormalities	5 (71%)	7 (100%)	0.462
Repeat CCT after 6 h	2 (29%)	3 (43%)	1.000
Repeat CCT after 24 h	–	2 (29%)	N/A
Intracranial lesions, combination	3 (43%)	6 (86%)	0.343
Anisocoria	1 (14%)	5 (71%)	0.103
Ventilation time	12 [6.0; 24]	192 [96; 336]	0.002
Circulatory support	1 (14%)	1 (14%)	1.000
Convulsions	1 (14%)	1 (14%)	1.000
Neurosurgery	–	1 (14%)	N/A
ICP monitoring	–	1 (14%)	N/A
Days with neuromonitoring	–	10 (*N* = 1)	N/A
Associated trauma	1 (14%)	5 (71%)	0.103
Mechanism of injury			0.001
-Fall	–	–	
-TA	7 (100%)	–	
	–	7 (100%)	

Data are presented as number (%) or median [IQR 25th–75th].

CCT, cerebral computed tomography; GCS/GMS, Glasgow coma scale/Glasgow motor scale; GCS/GMS aDP, GCS/GMS at discharge PICU; ICP, intracranial pressure; iGCS, initial GCS; iGMS, initial GMS; N*,* number; N/A, not applicable; NWT, neurological wake-up test; PICU, pediatric intensive care unit; PIM, pediatric index of mortality score; PRISM, pediatric risk of mortality [PRISMII] score; TA, traffic accident; y, year.

^a^ No statistics are computed because survival is a constant

Reasons for cessation of the NWT were non-arousal (71%) or severe agitation (29%). No clinical signs of increasing ICP during the NWT were observed. All but one of the NWTs in this subgroup were requested by the attending neurologist (Table 3). Subjects who failed the NWT subsequently had radiological examination (57%), request for a new NWT (14%), continuous interruption of sedation (14%) or intracranial pressure monitoring (14%). Although a significant difference (*p* = 0.036) in sedation use between both NWT-subgroups (successful vs. failure) was shown, both subgroups were considered comparable (Supplementary Table C-III) due to equal use of propofol with or without a combination of intravenous opioids and benzodiazepines, albeit in different combinations.

**Table 3:** NWT-results between NWT-success and NWT-failure group.

	NWT-outcome NWT-successful— NWT-failure	*p*-value
NWT-		
<24 h	7-7	^a^
Indication		*p* = 0.135
• Reasonable detubation • Degradation of GCS-score • Awareness	2-0 0–2 5-5	
Requested by		*p* = 0.012
• Neurologist • Neurosurgeon • PICU • Unknown	0–6 1-0 3-0 3-1	
Result		*p* = 0.001
• Not aroused • Prematurely terminated • Aroused	0–5 0–2 7-0	
Different policy afterwards?		*p* = 0.559
• No • Yes	6-4 1–3	
Treatment plan		*p* = 0.017
• Restarting sedation & CCT • Restarting sedation & new NWT • Detubation • Continuous sedation interruption • Restarting sedation & introducing ICPm	0–4 1-1 6-0 0–1 0–1	
Justifiable?		*p* = 0.266
• No, other parameters available • Yes	1–4 6-3	
Complications?		*p* = 0.005
• No; no change of parameters nor arousal • No, succesfully aroused • Yes, prematurely terminated	1–5 6-0 0–2	

CCT, cerebral computed tomography; GCS, Glasgow coma scale; ICPm, intracranial pressure monitoring; NWT, neurological wake-up test; PICU, pediatric intensive care unit.

^a^ No statistics are computed because NWT_24 h is a constant.

No significant complications as a result of the NWTs were observed due to cessation of the test when failure criteria were met.

### Non-NWT

Descriptive characteristics of the non-NWTs (*n* = 22) are summarized in Table 4. Patients with NWTs had less severe head injury marked by higher GCS/GMS scores upon admission (6–7 vs. 4–5; *p* = 0.045/4 vs. 2; *p* = 0.019) as well at discharge (13–14 vs. 9; *p* = 0.014/6 vs. 5; 0.070) and significant lower median PRISM (11 vs. 15.5; *p* = 0.014) and PIM scores (−3.1 vs. −2.5; *p* = 0.008). Patients without NWT more often required circulatory support (82% vs. 14%, *p* < 0.001), ICP-monitoring (45% vs. 7%, *p* = 0.025) or neurosurgical interventions (68% vs. 7%, *p* < 0.001) and they had higher mortality (59% vs. 100%, *p* = 0.006). Intracranial pressure monitoring was used in 11 (50%) of patients without NWT. All ICP-monitoring devices were placed within the first 24 h.

**Table 4:** NWT-outcome of NWT-group vs non-NWT group. Data are presented as number (%) or median (IQR 25th–75th).

	NWT	Non-NWT	*p*-value
Number	14	22	
Age			
Year	−8,5 [4¾–12]	−11 [6;14]	0.291
Month	–	−3 [1¼;5]	–
Gender, male	7 (50%)	14 (64%)	0.418
PRISM	11 [5½;15½]	15,5 [12,8;25½]	0.014
PIM	−3,1 [−3,2;−2,9]	−2,5 [−3;−0,9]	0.008
iGCS	6–7 [4;7]	4–5 [3;7]	0.045
iGMS	4 [2;4]	2 [1;4]	0.019
GCS aDP	13–14 [12;15]	9 [3;14]	0.014
GMS aDP	6 [5;6]	5 [1;6]	0.070
Survival	14 (100%)	13 (59%)	0.006
Initial CCT-scan abnormalities	12 (86%)	21 (95%)	0.547
Repeat CCT after 6 h	5 (36%)	7 (32%)	0.809
Repeat CCT after 24 h	2 (14%)	6 (27%)	0.441
Intracranial lesions			0.128
Contusion only	−3	−1	
SDH	−0	−2	
SAH	−1	−0	
IPH	−1	−0	
Combination	−9	−19	
Ventilation time	48 [11;198]	168 [24;240]	0.283
Circulatory support	2 (14%)	18 (82%)	0.000
Convulsions	2 (14%)	4 (18%)	1.000
Neurosurgery	1 (7%)	15 (68%)	0.000
ICP monitoring	1 (7%)	10 (45%)	0.025
Days with neuromonitoring	[10, *N *= 1]	6 [4;10] (*N* = 11)	0.015
Associated trauma	6 (43)	16 (73%)	0.073
MoI			0.057
Fall	−7	−3	
TA	−7	−14	
NAI	−0	−3	
Other	−0	−2	

CCT, cerebral computed tomography; GCS/GMS, glasgow coma scale/glasgow motor scale; GCS/GMS aDP, GCS/GMS at discharge PICU; ICP, intracranial pressure; iGCS, initial GCS; iGMS, initial GMS; IPH, intraparenchymal hematoma; m, months; N, number; NAI, non-accidental injury; NWT, neurological wake-up test; PICU, pediatric intensive care unit; PIM, pediatric index of mortality score; PRISM, pediatric RISk of mortality [PRISMII] score; SAH, subarachnoid hemorrhage; SDH, subdural hematoma; TA, traffic accident; y, year.

Patients without NWTs had a significantly higher PIM score (−2.5 vs. −3.1, *p* = 0.017), and more often required circulatory support (82% vs. 14%, *p* = 0.003) and neurosurgical intervention (68% vs. 14%, *p* = 0.026) compared to the patients in whom the NWT was defined as failed (Table 5). GCS/GMS scores upon admission and discharge and initial CCT-scan abnormalities between non-NWTs and patients in whom the NWT failed were comparable, as were age, ventilation time, associated trauma and underlying mechanism of injury. The successful NWTs were less similar to the non-NWTs (Supplementary Table D): patients without NWT had significantly longer ventilation time (168 vs.12 h, *p* = 0.004), more circulatory support (82% vs. 14%, *p* = 0.003), intracranial lesions (82% vs. 43%, *p* = 0.045), neurosurgery (68% vs. 0%, *p* = 0.002), associated trauma (73% vs. 14%, *p* = 0.011) and underlying mechanism of injury (several mechanisms vs. 100% fall, *p* = 0.001) (Supplementary Table E).

**Table 5:** NWT-outcome of NWT-failure group vs non-NWT group. Data are presented as number (%) or median (IQR 25th–75th).

	NWT-failure	Non-NWT	*p*-value
Number	7	22	
Age			
Year	−12[9;14]	−11 [6;14]	0.588
Month		−3 [1¼;5]	
Gender, male	5 (71%)	14 (64%)	1.000
PRISM	13 [5;17]	15,5 [12,8;25,5]	0.160
PIM	−3,1 [−3,4;−2,9]	−2,5 [−3;−0,9]	0.017
iGCS	6 [4;7]	4–5 [3;7]	0.210
iGMS	4 [2;5]	2 [1;4]	0.070
GCS aDP	12 [9;12]	9 [3;14]	0.310
GMS aDP	5 [4;6]	5 [1;6]	0.628
Survival	7 (100%)	13 (59%)	0.066
Initial CCT-scan abnormalities	7 (100%)	21 (96%)	1.000
Repeat CCT after 6 h	3 (43%)	7 (32%)	0.665
Repeat CCT after 24 h	2 (29%)	6 (27%)	1.000
Intracranial lesions	6 (86%)	18 (82%)	0.638
Anisocoria	5 (71%)	16 (73%)	0.215
Ventilation time	192 [96;336]	168 [24;240]	0.230
Circulatory support	1 (14%)	18 (82%)	0.003
Convulsions	1 (14%)	4 (18%)	1.000
Neurosurgery	1 (14%)	15 (68%)	0.026
ICP monitoring	1 (14%)	11 (50%)	0.202
Days with neuromonitoring	10 (*N* = 1)	6 [4;10] (*N *= 11)	0.162
Associated trauma	5 (71%)	16 (73%)	1.000
Mechanism of injury			0.319
Fall	–	−3 (14%)	
TA	−7 (100%)	−14 (64%)	
NAI	–	−3 (14%)	
Other	–	−2 (9%)	

CCT, cerebral computed tomography; GCS/GMS, glasgow coma scale/glasgow motor scale; GCS/GMS aDP, GCS/GMS at discharge PICU; ICP, intracranial pressure; iGCS, initial GCS; iGMS, initial GMS; m, months; N, number; NAI, non-accidental injury; NWT, neurological wake-up test; PICU, pediatric intensive care unit; PIM, Pediatric index of mortality score; PRISM, pediatric RISk of mortality [PRISMII] score; TA, traffic accident; y, year.

In two patients the NWT was not performed, despite being requested. Reasons for not doing NWTs were severe trauma requiring neurosurgical interventions and refractory intracranial hypertension (ICH). No NWT was requested in the remaining 20 patients, mainly because of ICH (62%).

In four patients (19%) we could not retrieve the rationale for not performing the NWT in the first 24 h. Patients without NWTs received significant more long half-life sedatives and more often a combination of several sedatives when compared to the patients with NWTs (*p* = 0.003, Supplementary Table C-II).

### Outcome

Nine patients (25%) died during their PICU admission, none of them had an NWT. Three of them were younger than 6 months. Fourteen patients were ventilated <24 h, with seven of them having a complete NWT. The other seven patients died. Five of them had TA, 2 non-accidental injury (NAI), 1 other and 1 fall from height.

## Discussion

To our best knowledge, this is the first study reporting the feasibility of the neurological wake-up test (NWT) in critically ill children with severe TBI. In the described cohort, NWTs were infrequently performed (mainly in those with less severe manifestations of the pTBI) and were successful in about half of these patients.

NWTs are considered gold standard in the evaluation of patients with severe TBI, despite the fact that NWTs are being absent in international (pediatric) guidelines. However, we found that NWTs were performed in less than half of our cohort. Strikingly, no NWTs were performed in children <6 months of age. In retrospect, no clear explanation could be identified. One of the possible reasons for a low use of NWTs might be the confusion about terminology. It has been proposed that the response to sedation interruption should be addressed as “arousal” and not to “awakening” (22). As such, the term “wake-up test” is confusing and this could result in different predetermined goals. As Table 1 shows, there was a difference in predetermined goals in our NWT-group. When a reasonable detubation was considered, the NWT was mostly suggested by the pediatric intensivist. In the other cases, “awareness” and “deterioration” were the indications by the neurologist. This emphasizes the importance of speaking the same language in a multidisciplinary team. Another explanation could be that performing the NWT requires an immediate disruption in administration of sedato-analgesic drugs. This may be a barrier particularly in the PICU because in general there is little use of sedato-analgesic drugs with short half-lives and a low potential of inducing tolerance and withdrawal symptoms, which will hamper the NWT (23). Short-acting sedatives such as propofol are contra-indicated in younger children, resulting in the use of drugs with a relatively long half-life (8).

Interestingly, while all patients with NWT-success had an initial GCS ≤8, they were extubated very rapidly after completing the NWT. No doubt, these patients benefited from the NWT, but it might also be surmised that the initial GCS assessment was incorrect. It is known that about 20% of TBI patients are misclassified as severe (4, 24). We found no differences in use of sedatives in the NWT-group, thereby not explaining the non-arousal in the failure-group. The frequent use of propofol in both NWT-groups suggests that these patients were deemed to have less severe brain injury based on the higher initial GCS/GMS scores upon PICU admission and lower PRISM II score.

Importantly, there was no standardized protocol on when or how to use the NWT. Therefore, the actual performance of the NWT was dependent on the discretion of the attending physician. No time-interval of sedation interruption to clinical evaluation was defined and this could not be distracted from patient records. This may have resulted in premature ending of the NWT, classifying it as a failure. Hence, our study suffers from confounding by indication. It may be surmised that NWTs were not performed in patients with more severe brain injury. We observed no differences in clinical characteristics between patients without NWT and those in whom the NWT failed except for higher PRISM/PIM scores, more additional trauma and circulatory support and more invasive monitoring. All non-survivors were non-NWTs. Therefore, we cannot rule out a clinical preference for not doing NWTs in those with the most severe brain injury because of the supposed risks such as increases in ICP. While awaiting arousal a variable response can occur due to ongoing pharmacological effects and alterations in behavior resulting from brain damage (22). The NWT procedure induces a biochemical response and short duration of increase in ICP and CPP (11–13). Agitation is a common finding in TBI patients and is associated with poor functional outcome depending on severity and duration of the agitation (25). Pulmonary edema has also been described as a consequence of prolonged agitation (26). Recurrent agitation as a result of frequent NWT-trials therefore should be prevented.

Further studies are indicated to identify patients in whom the NWT is indicated. We observed some interesting differences between the NWT-successes vs. failures which might guide future decision-making. We found differences in age and mechanism of injury. Furthermore, NWT-failures had a significant lower GCS/GMS score at discharge. All 7 NWT-failures had high impact TAs (mean velocity 75 km/h) caused by pedestrian (*n* = 1) or (non-helmeted) cyclist (*n* = 6) vs. car collision and all of them showed ongoing reduced levels of consciousness (GCS <8) and were diagnosed as having diffuse axonal injury (DAI). It may therefore be postulated that vulnerable road users (VRU's) (pedestrians, cyclists e.g.,) with a known high impact TA and consequently risk of DAI in the absence of focal deficits should not be selected for the NWT (27, 28). Patients in this group do not benefit from the NWT within the first 24 h and clinical evaluations that could cause secondary insults should be postponed. In this setting, the clinician should rely on neuroimaging and multimodality monitoring (18, 29).

Our study had several additional limitations. First, our study was designed as a single center retrospective study, limiting generalizability of our findings. Second, we have studied a heterogeneous population with a small total number of included patients. Third, there was no standardized protocol for performing the NWT. Fourth, limited data concerning the involvement of CCT interpretation in performing NWTs could be retrieved from our data (30). Therefore, we do not know to what extent the decision to perform NWTs or not was influenced by this interpretation or by the clinical presentation at onset. Such information should be incorporated into future studies. Nonetheless, our data can be interpreted as a small but hopefully important contribution in gaining knowledge regarding NWTs in critically ill children with severe TBI.

## Conclusion

We observed a low use of NWTs in children with severe TBI. Patients in whom the NWT was successful had relatively low-impact injury and where extubated very rapidly after the NWT. Those in whom the NWT-failed showed similar characteristics to those in whom an NWT was not performed. As many factors may play a role in the success and yield of an NWT, the decision to perform this test should be based on multidisciplinary evaluation. The timing of the NWT within the first 24 h of admission seems appropriate, as it may expose those patients initially misclassified as severe. Although no complications were observed, no conclusions can be made about the safety of the NWT based on our results.

Further research is much needed to truly establish the feasibility, safety of and indications for the NWT in severe pTBI. A future prospective study should not only include factors such as age, GCS-score at onset or mechanism of injury, but also include CCT-interpretations (Rotterdam and Marshall score) to determine in which subgroups of patients an NWT is of value.

## Data Availability

The original contributions presented in the study are included in the article/Supplementary Material, further inquiries can be directed to the corresponding author.
