# Supplementary material for: The neurological wake-up test in severe pediatric traumatic brain injury: a long term, single-center experience
Source: Front Pediatr. 2024 Feb 23;12:1367337. doi: 10.3389/fped.2024.1367337 (PMC10920253; doi:10.3389/fped.2024.1367337)
Supplement: Supplementary file 1 [file Table1.docx]

| **Patient** | **Age** | **Sex** | **TM** | **iGCS (iGMS)** | **NWT** | **Year** | **Survival** | **GCS (GMS) aDP** | **ICP/EVD** |
| --- | --- | --- | --- | --- | --- | --- | --- | --- | --- |
| 1 | 14 y | M | TA | 5 (3) | No | 2014 | Yes | 10 (4) | No |
| 2 | 12 y | F | Fall | 5 (3) | Yes | 2014 | Yes | 12 (6) | No |
| 3 | 02 y | M | Fall | 4 (2) | Yes | 2014 | Yes | 15 (6) | No |
| 4 | 08 y | M | TA | 4 (2) | Yes | 2013 | Yes | 12 (6) | No |
| 5 | 16 y | M | TA | 6 (4) | No | 2015 | Yes | 14 (6) | Yes |
| 6 | 09 y | M | TA | 4 (2) | Yes | 2014 | Yes | 12 (5) | No |
| 7 | 08 y | M | TA | 4 (2) | No | 2014 | No | 03 (1) | Yes |
| 8 | 07 y | M | TA | 3 (1) | No | 2011 | Yes | 15 (6) | Yes |
| 9 | 15 y | M | Other | 3 (1) | No | 2014 | No | 03 (1) | Yes |
| 10 | 06 y | F | Fall | 8 (4) | Yes | 2012 | Yes | 15 (6) | No |
| 11 | 02 m | M | NAI | 3 (1) | No | 2011 | No | 03 (1) | No |
| 12 | 12 y | M | TA | 7 (5) | Yes | 2015 | Yes | 12 (5) | Yes |
| 13 | 13 y | F | TA | 7 (3) | No | 2016 | Yes | 08 (5) | No |
| 14 | 06 m | M | NAI | 3 (1) | No | 2014 | No | 03 (1) | No |
| 15 | 15 y | M | TA | 4 (2) | Yes | 2015 | Yes | 12 (5) | No |
| 16 | 05 y | M | TA | 3 (1) | No | 2014 | No | 03 (1) | No |
| 17 | 02 y | F | Fall | 7 (4) | Yes | 2015 | Yes | 15 (6) | No |
| 18 | 05 y | F | Fall | 8 (4) | Yes | 2016 | Yes | 15 (6) | No |
| 19 | 05 y | F | Other | 6 (3) | No | 2015 | Yes | 13 (6) | Yes |
| 20 | 03 m | M | NAI | 6 (2) | No | 2012 | Yes | 15 (6) | Yes |
| 21 | 04 y | F | Fall | 6 (3) | Yes | 2012 | Yes | 15 (6) | No |
| 22 | 11 y | M | TA | 8 (5) | Yes | 2017 | Yes | 08 (4) | No |
| 23 | 05 m | M | Fall | 8 (4) | No | 2015 | Yes | 15 (6) | No |
| 24 | 12 y | F | TA | 7 (4) | Yes | 2014 | Yes | 09 (4) | No |
| 25 | 13 y | F | TA | 7 (4) | No | 2017 | Yes | 11 (6) | Yes |
| 26 | 15 y | F | TA | 6 (4) | No | 2015 | Yes | 13 (6) | Yes |
| 27 | 12 y | M | TA | 3 (1) | No | 2018 | Yes | 15 (6) | No |
| 28 | 14 y | F | TA | 6 (4) | Yes | 2013 | Yes | 15 (6) | No |
| 29 | 14 y | F | TA | 4 (2) | No | 2018 | Yes | 11 (6) | Yes |
| 30 | 02 y | M | TA | 7 (4) | No | 2018 | Yes | 15 (6) | No |
| 31 | 04 m | F | Fall | 8 (3) | No | 2020 | No | 03 (1) | No |
| 32 | 09 y | F | Fall | 7 (5) | No | 2020 | Yes | 08 (5) | Yes |
| 33 | 05 y | M | Fall | 7 (4) | Yes | 2020 | Yes | 15 (6) | No |
| 34 | 01 y | F | TA | 3 (1) | No | 2020 | No | 03 (1) | No |
| 35 | 09 y | M | TA | 3 (1) | No | 2020 | No | 03 (1) | Yes |
| 36 | 11 y | M | TA | 3 (1) | No | 2020 | No | 03 (1) | No |

**Table A.** Baseline characteristics; *EVD=external ventricular drain; F=female; GCS (GMS) aDP= Glasgow coma scale (Glasgow motor scale) at discharge PICU; ICP=intracranial pressure monitoring; iGCS (iGMS)= initial GCS (GMS); m=months; M=male; MoI=mechanism of injury; NAI=non-accidental injury, NWT=neurological wake-up test*; *TA=traffic accident; y=years*
