# Supplementary material for: The neurological wake-up test in severe pediatric traumatic brain injury: a long term, single-center experience
Source: Front Pediatr. 2024 Feb 23;12:1367337. doi: 10.3389/fped.2024.1367337 (PMC10920253; doi:10.3389/fped.2024.1367337)
Supplement: Supplementary file 2 [file Table2.docx]

|  | **No of patients** | **NWT** | **Survival** |
| --- | --- | --- | --- |
| 2011 | 2 | 0 [ 0%] | 1 [ 50%] |
| 2012 | 3 | 2 [66%] | 3 [100%] |
| 2013 | 2 | 1 [50%] | 2 [100%] |
| 2014 | 9 | 4 [44%] | 5 [ 56%] |
| 2015 | 7 | 2 [29%] | 7 [100%] |
| 2016 | 2 | 1 [50%] | 2 [100%] |
| 2017 | 2 | 1 [50%] | 2 [100%] |
| 2018 | 3 | 0 [ 0%] | 3 [100%] |
| 2020 | 6 | 1 [17%] | 2 [ 33%] |

**Table B**. NWTs during study period. [of notice: there were no patients with a GCS <8 in 2019] *No= number; NWT=neurological wake-up test*
