# Supplementary material for: The neurological wake-up test in severe pediatric traumatic brain injury: a long term, single-center experience
Source: Front Pediatr. 2024 Feb 23;12:1367337. doi: 10.3389/fped.2024.1367337 (PMC10920253; doi:10.3389/fped.2024.1367337)
Supplement: Supplementary file 3 [file Table3.docx]

| *Table C-I*  Sedation | Frequency | % | Valid% | Cumulative% |
| --- | --- | --- | --- | --- |
| Midazolam/Morphine | 5 | 13.9 | 13.9 | 13.9 |
| Midazolam/Fentanyl | 3 | 8.3 | 8.3 | 22.2 |
| Propofol | 4 | 11.1 | 11.1 | 33.3 |
| Propofol/Morphine | 11 | 30.6 | 60.6 | 63.9 |
| Other combination | 13 | 36.1 | 36.1 | 100.0 |
| Total | 36 | 100 | 100 | 100 |

| *Table C-II*  Sedation | No NWT | NWT-success | NWT-failure | Total | p-value |
| --- | --- | --- | --- | --- | --- |
| Midazolam/Morphine | 3 | 2 | 0 | 5 |  |
| Midazolam/Fentanyl | 3 | 0 | 0 | 3 |  |
| Propofol | 1 | 3 | 0 | 4 |  |
| Propofol/Morphine | 4 | 1 | 6 | 11 |  |
| Other combination | 11 | 1 | 1 | 13 |  |
| Total | 22 | 7 | 7 | 36 | p=0.003 |

| *Table C-III*  Sedation | NWT-success | NWT-failure | Total | p-value |
| --- | --- | --- | --- | --- |
| Midazolam/Morphine | 2 | 0 | 5 |  |
| Midazolam/Fentanyl | 0 | 0 | 3 |  |
| Propofol | 3 | 0 | 4 |  |
| Propofol/Morphine | 1 | 6 | 11 |  |
| Other combination | 1 | 1 | 13 |  |
| Total | 7 | 7 | 36 | p=0.036 |

**Table C.** Overall sedation use versus NWT-outcome; I-III. *NWT=neurological wake-up test*
