# Supplementary material for: The neurological wake-up test in severe pediatric traumatic brain injury: a long term, single-center experience
Source: Front Pediatr. 2024 Feb 23;12:1367337. doi: 10.3389/fped.2024.1367337 (PMC10920253; doi:10.3389/fped.2024.1367337)
Supplement: Supplementary file 4 [file Table4.docx]

|  | **NWT** | **Non-NWT** | **p-value** |
| --- | --- | --- | --- |
| N | 14 | 22 |  |
| Age  -y  -m | -8,5 [4¾ -12]  -- | -11[6;14]  - 3[1¼;5] | 0,291  -- |
| Gender, male | 7 (50%) | 14 (64%) | 0,418 |
| PRISM | 11 [5½ ;15½] | 15,5 [12,8;25½ ] | 0,014 |
| PIM | -3,1 [-3,2;-2,9] | -2,5 [-3;-0,9] | 0,008 |
| iGCS | 6-7 [4;7] | 4-5 [3;7] | 0,045 |
| iGMS | 4 [2;4] | 2 [1;4] | 0,019 |
| GCS aDP | 13-14 [12;15] | 9 [3;14] | 0,014 |
| GMS aDP | 6 [5;6] | 5 [1;6] | 0,070 |
| Survival | 14 (100%) | 13 (59%) | 0,006 |
| Initial CCT-scan abnormalities | 12 (86%) | 21 (95%) | 0,547 |
| Repeat CCT after 6 hours | 5 (36%) | 7 (32%) | 0,809 |
| Repeat CCT after 24 hours | 2 (14%) | 6 (27%) | 0,441 |
| Intracranial lesions  -contusion only  -SDH  -SAH  -IPH  -combination | -3  -0  -1  -1  -9 | -1  -2  -0  -0  -19 | 0,128 |
| Ventilation time | 48 [11;198] | 168 [24;240] | 0,283 |
| Circulatory support | 2 (14%) | 18 (82%) | 0,000 |
| Convulsions | 2 (14%) | 4 (18%) | 1,000 |
| Neurosurgery | 1 (7%) | 15 (68%) | 0,000 |
| ICP monitoring | 1 (7%) | 10 (45%) | 0,025 |
| Days with neuromonitoring | [10, N=1] | 6 [4;10] (N=11) | 0,015 |
| Associated trauma | 6 (43) | 16 (73%) | 0,073 |
| MoI  -fall  -TA  -NAI  -other | -7  -7  -0  -0 | -3  -14  -3  -2 | 0,057 |

**Table 4.** NWT-outcome of NWT-group vs non-NWT group. Data are presented as number (%) or median (IQR 25^th^-75^th^). *CCT= cerebral computed tomography; GCS/GMS= Glasgow coma scale/ Glasgow motor scale; GCS/GMS aDP= GCS/GMS at discharge PICU; ICP= intracranial pressure; iGCS=initial GCS; iGMS=initial GMS; IPH=intraparenchymal hematoma; m=months; N=number; NAI=non-accidental injury; NWT= neurological wake-up test; PICU= pediatric intensive care unit; PIM= Pediatric Index of Mortality score; PRISM =Pediatric RISk of Mortality [PRISMII] score; SAH=subarachnoid hemorrhage; SDH=subdural hematoma; TA=traffic accident; y=year*
