# Supplementary material for: The neurological wake-up test in severe pediatric traumatic brain injury: a long term, single-center experience
Source: Front Pediatr. 2024 Feb 23;12:1367337. doi: 10.3389/fped.2024.1367337 (PMC10920253; doi:10.3389/fped.2024.1367337)
Supplement: Supplementary file 5 [file Table5.docx]

|  | **NWT-failure** | **Non-NWT** | **p-value** |
| --- | --- | --- | --- |
| N | 7 | 22 |  |
| Age  -y  -m | -12[9;14] | -11[6;14]  - 3[1¼;5] | 0,588 |
| Gender, male | 5 (71%) | 14 (64%) | 1,000 |
| PRISM | 13 [5;17] | 15,5 [12,8;25,5] | 0,160 |
| PIM | -3,1 [-3,4;-2,9] | -2,5 [-3;-0,9] | 0,017 |
| iGCS | 6 [4;7] | 4-5 [3;7] | 0,210 |
| iGMS | 4 [2;5] | 2 [1;4] | 0,070 |
| GCS aDP | 12 [9;12] | 9 [3;14] | 0,310 |
| GMS aDP | 5 [4;6] | 5 [1;6] | 0,628 |
| Survival | 7 (100%) | 13 (59%) | 0,066 |
| Initial CCT-scan abnormalities | 7 (100%) | 21 (96%) | 1,000 |
| Repeat CCT after 6 hours | 3 (43%) | 7 (32%) | 0,665 |
| Repeat CCT after 24 hours | 2 (29%) | 6 (27%) | 1,000 |
| Intracranial lesions | 6 (86%) | 18 (82%) | 0,638 |
| Anisocoria | 5 (71%) | 16 (73%) | 0,215 |
| Ventilation time | 192 [96;336] | 168 [24;240] | 0,230 |
| Circulatory support | 1 (14%) | 18 (82%) | 0,003 |
| Convulsions | 1 (14%) | 4 (18%) | 1,000 |
| Neurosurgery | 1 (14%) | 15 (68%) | 0,026 |
| ICP monitoring | 1 (14%) | 11 (50%) | 0,202 |
| Days with neuromonitoring | 10 (N=1) | 6 [4;10] (N=11) | 0,162 |
| Associated trauma | 5 (71%) | 16 (73%) | 1,000 |
| Mechanism of injury  -fall  -TA  -NAI  -other | --  -7 (100%)  --  -- | -3 (14%)  -14 (64%)  -3 (14%)  -2 (9%) | 0,319 |

**Table 5.** NWT-outcome of NWT-failure group vs non-NWT group. Data are presented as number (%) or median (IQR 25^th^-75^th^). *CCT= cerebral computed tomography; GCS/GMS= Glasgow coma scale/ Glasgow motor scale; GCS/GMS aDP= GCS/GMS at discharge PICU; ICP= intracranial pressure; iGCS=initial GCS; iGMS=initial GMS; m=months; N=number; NAI=non-accidental injury; NWT= neurological wake-up test; PICU= pediatric intensive care unit; PIM= Pediatric Index of Mortality score; PRISM =Pediatric RISk of Mortality [PRISMII] score; TA=traffic accident; y=year*
