# Supplementary material for: The neurological wake-up test in severe pediatric traumatic brain injury: a long term, single-center experience
Source: Front Pediatr. 2024 Feb 23;12:1367337. doi: 10.3389/fped.2024.1367337 (PMC10920253; doi:10.3389/fped.2024.1367337)
Supplement: Supplementary file 6 [file Table6.docx]

|  | **ICP/EVD [Total no of pt]** | **MoI** | **Survival** |
| --- | --- | --- | --- |
| 2011 | 1 [2/50%] | TA | 1 [ 50%] |
| 2012 | 1 [3/33%] | NAI | 3 [100%] |
| 2013 | 0 [2/ 0%] | - | 2 [100%] |
| 2014 | 2 [9/22%] | TA, other | 5 [ 56%] |
| 2015 | 4 [7/57%] | TA [3], other | 7 [100%] |
| 2016 | 0 [2/ 0%] | - | 2 [100%] |
| 2017 | 1 [2/50%] | TA | 2 [100%] |
| 2018 | 1 [3/33%] | TA | 3 [100%] |
| 2020 | 2 [6/33%] | TA, fall | 2 [ 33%] |

**Table F**. ICP monitoring during study period. *EVD=external ventricular drain; ICP= intracranial pressure; MoI=mechanism of injury; NAI= non accidental injury No= number; pt=patients; TA=traffic accident*
