# Supplementary material for: The neurological wake-up test in severe pediatric traumatic brain injury: a long term, single-center experience
Source: Front Pediatr. 2024 Feb 23;12:1367337. doi: 10.3389/fped.2024.1367337 (PMC10920253; doi:10.3389/fped.2024.1367337)
Supplement: Supplementary file 7 [file Table7.docx]

| Patient [N=14] | MV ≤ 24 hours [in hours] | MoI | NWT | Survival | iGCS (GMS) |
| --- | --- | --- | --- | --- | --- |
| 2 | 24 | Fall | Yes | Yes | 5 () |
| 3 | 12 | Fall | Yes | Yes | 4 () |
| 7 | 24 | TA | No | No | 4 () |
| 10 | 24 | Fall | Yes | Yes | 8 () |
| 14 | 24 | NAI | No | No | 3 (1) |
| 16 | 12 | TA | No | No | 3 (1) |
| 17 | 3 | Fall | Yes | Yes | 7 () |
| 18 | 8 | Fall | Yes | Yes | 8 () |
| 21 | 6 | Fall | Yes | Yes | 6 () |
| 31 | 12 | Fall | No | No | 8 () |
| 33 | 12 | Fall | Yes | Yes | 7 () |
| 34 | 4 | TA | No | No | 3 (1) |
| 35 | 6 | TA | No | No | 3 (1) |
| 36 | 24 | TA | No | No | 3 (1) |

**Table G.** Mechanical ventilation ≤ 24 hours in accordance to underlying traumatic mechanism, [non-]NWT, survival and initial GCS. *iGCS= initial Glasgow Coma Scale; GMS = Glasgow Motor Scale; MoI=mechanism of injury; MV=mechanical ventilation; N=number; NAI= non accidental injury; TA= traffic accident*
